# Evaluation of Patient Safety Indicators in Semnan City Hospitals by Using the Patient Safety Friendly Hospital Initiative (PSFHI)

**DOI:** 10.5539/gjhs.v8n8p1

**Published:** 2015-12-17

**Authors:** Hassan Babamohamadi, Roghayeh Khabiri Nemati, Monir Nobahar, Seifullah Keighobady, Soheila Ghazavi, Farideh Izadi-Sabet, Zhila Najafpour

**Affiliations:** 1 School of Nursing and Allied Health, Semnan University of Medical Sciences (SemUMS), Semnan, Iran; 2 National Institute of Health Research, Tehran University of Medical Sciences (TUMS), Tehran, Iran; 3 School of Public Health, Tehran University of Medical Sciences (TUMS), Tehran, Iran

**Keywords:** patient safety friendly hospital initiative (PSFHI), Iranian hospitals, safety standards

## Abstract

**Background:**

Nowadays, patient safety issue is among one of the main concerns of the hospital policy worldwide. This study aimed to evaluate the patient safety status in hospitals affiliated to Semnan city, using the WHO model for Patient Safety Friendly Hospital Initiatives (PSFHI) in summer 2014.

**Methods:**

That was a cross sectional descriptive study that addressed patient safety, which explained the current status of safety in the Semnan hospitals using by instrument of Patient safety friendly initiative standards (PSFHI). Data was collected from 5 hospitals in Semnan city during four weeks in May 2014.

**Results:**

The finding of 5 areas examined showed that some components in critical standards had disadvantages. Critical standards of hospitals including areas of leadership and administration, patient and public involvement and safe evidence-based clinical practice, safe environment with and lifetime education in a safe and secure environment were analyzed. The domain of patient and public involvement obtained the lowest mean score and the domain of safe environment obtained the highest mean score in the surveyed hospitals.

**Conclusion:**

All the surveyed hospitals had a poor condition regarding standards based on patient safety. Further, the identified weak points are almost the same in the hospitals. Therefore, In order to achieve a good level of all aspects of the protocol, the goals should be considered in the level of strategic planning at hospitals. An effective execution of patient safety creatively may depend on the legal infrastructure and enforcement of standards by hospital management, organizational liability to expectation of patients, safety culture in hospitals.

## 1. Introduction

### 1.1 Introduce the Problem

Following the Institute of Medicine (IO report which revealed the extent of harm caused by healthcare, patient safety was put on the policy agenda of healthcare systems worldwide (Kohn et al., 2000; Evanoff et al., 2005). Several studies confirmed that alongside the enormous benefit of medical care, they have significant risks to patients (2011b).

Researches supporting evidence on patient safety is currently little in developing countries and i countries with economies in the transfer (Aghaei et al., 2014) and also there is a few issued evidence from Iran that estimates the range of adverse events in health care settings (Moghri et al., 2012).

Patient safety is a critical subject of health care quality. Today’s patient safety is a priority for all health care systems that strive to ensure patient safety and meliorate quality of care. Patient safety is introduced as the avoidance, prevention and correction of damages and adverse consequences of the health care process (Edwin, 2009).

Also medical errors can be introduced as the failure of a planned action to be completed as intended or the use of a wrong plan to achieve an aim. Types of errors are diagnostic, treatment, preventive and other (failure of communication, equipment failure and other system failure (2011a).

Although the concept of achieving the efficient improvement in healthcare remains to be ideally understood, hospitals are responsible for strengthening the health system and this may be accomplished only through quality improvement (Bates et al., 2001; Bates et al., 2003).

Adverse events and medical errors are challenges that health system of all countries face them and strive to minimize them. One fifth of the population is at risk of medical errors leading to die or suffer from preventable injuries (Abdi & Maleki, 2011).

Studies show that 3 to 17 percent of patients admitted to hospitals suffer from harm or complications that were caused by adverse events or medical errors and estimates about 30-70 percent of events are preventable (Classen et al., 2011).

These events lead to damaging about 8500000 people in year and losses to the UK health system (NHS) about 1 to 2 milliard pounds (Johnstone & Kanitsaki, 2007).

Health care at the United States at least 44,000 people, and probably as many as 98,000 people, die in hospitals each year as a due to medical errors that could have been prevented, according to appraises from two major studies [references]. Even using the lower appraise, preventable medical errors in hospitals exceed ascribable deaths to such scary threats as motor-vehicle wrecks, breast cancer, and AIDS (Reynard et al., 2009).

However, there is a growing movement started by patients for complaints against healthcare personnel, particularly physicians, due to medical errors (Akbari et al., 2007).

This study aimed to evaluate the patient safety status in hospitals affiliated to Semnan medical university, using the WHO model for Patient Safety Friendly Hospital Initiatives (PSFHI) in summer 2014.

## 2. Methods

### 2.1 Study Design

This study was a cross sectional descriptive study that addressed patient safety, which explains the current status of safety in the Semnan hospitals. The study population was included all hospitals affiliated with Semnan University of Medical Sciences: Amir almomenin, Kawsar, 15 Khordad of Mehdishahr, Imam Khomeini of Garmsar, Velayat of Damghan and Shafa (Social Security).

There are several methods to measure the patient safety of which the instrument of Patient safety friendly initiative standards (PSFHI) is relatively common. This study used the Iranian version of this protocol that was confirmed by Ministry of Health and Medical Education (MOHME). Table 1 shows criteria for patient safety according to the WHO protocol.

**Table 1:** Domains and contributing standards

Domains	Critical standards	Corel standards	Developmental standards	Standards in each domain
A. Leadership and management (6 subdomains: A1-A)	9	20	7	36
B. Patient and public involvement (7 subdomains: B1-B7)	2	16	10	28
C. Safe evidence-based clinical practice (6 subdomains: C1-C6)	7	29	8	44
D. Safe environment (2 subdomains: D1-D2)	2	19	0	21
E. Lifelong learning (3 subdomains: E1-E3)	0	6	5	11
Total	20	90	30	140

The data collection was performed during four weeks in May 2014 in five hospitals in Semnan city (Iran).

### 2.2 Study Instrument

The Patient Safety Friendly Hospital Initiative (PSFHI) has five main groups of standards, which are divided into 24 subgroups. The five main groups are as follows: governance and leadership, participation and interaction with patients and the community, safe and evidence-based clinical services, secure environment and continuing education. The 5 main groups encompass 24 subgroups. The five main groups are classified into 3 categories: mandatory standards (a total of 20 standards), major standards (A total of 90 standard) and advanced standards (A total of 30 standards). Mandatory standards are needed to be fully (100%) met by candidate hospital Basic standards include mandatory standards with which a hospital has to coincident to become enrolled in the patient safety friendly hospital initiative. It is not mandatory for a hospital to meet 100% of the major standards in order to be enrolled in the patient safety friendly hospital initiative. Altogether, the percentage of the standards complied with will determine the level the hospital achieves.

Advanced standards, are requirements which a hospital depending on its capacity and resources must proceed to achieve them to improve the level of patient safety. The altogether approach adopted by the PSFHI has an assessment phase followed by a betterment phase. This study aimed to evaluate the patient safety status in hospitals affiliated to Semnan Medical University, using the WHO model for Patient Safety Friendly Hospital Initiatives (PSFHI) in summer 2014.

Countries of the Eastern Mediterranean Zone with patronage of the World Health Organization, have adopted a comprehensive approach to tackle the problem of unsafe healthcare in the zone through launching the Patient Safety Friendly Hospital Initiative (PSFHI). This initiative was launched by the Eastern Mediterranean Regional Office of the World Health Organization (WHO EMRO) in 2007. The main purpose of the PSFHI is to improve patient safety by developing harmonized standards to which hospitals adhere. PSFHI aims to encourage the participation of hospital managers and staff to cooperate in this effort. In addition, PSFHI encourages participation of national health authorities such as Ministries of Health, medical associations, and medical schools in the procedure of safe health care delivery and acts to potentiate and support global, regional and global health care accreditation plans.

Data was collected through interviews with different people depending on areas under investigation, observation and documentation.

Researchers attended in the field of hospitals and completed protocols based on the checklist guideline.

In the first step (before the interview), investigators randomly visited inpatient wards, intensive care units, emergency, pharmacy, sterilization centers, outpatient clinics, dental clinics, operating rooms, medical records department, radiology and laboratory, blood banks, kitchens and hospital waste temporary location.

Then, the interviews conducted with board of director, hospital manager, chief medical group, officer of the patient safety, senior pharmacist, medical devices engineer, head of health promotion, 3 nurses, 3 patients, blood bank manager, officer of medical records, expert on environmental health and work, infection control expert, doctor, hospital waste manager, staff professional promotion programs coordinator.

### 2.3 Statistical Analysis

All data were analyzed with SPSS software package, version 16.0. When analyzing, in terms of hospital being qualified in aspect of structure, process, output, is rated 1score, the relative score is 5.0 and not qualified is 0 point. Two two statistical tests were conducted to analyses the data: A Kruskal-Wallis test (the nonparametric version of the Analysis of Variance) and conceptual statistical method (single-sample **t**-test). All analyses are presented 95% confidence intervals.

### 2.4 Ethics

This study was done in line with the Declaration of Helsinki, and was approved by the ethics committee in Semnan University of Medical Sciences (reference no: 92/412574).

## 3. Results

In this section, the status of each hospital’s domains and standards were studied separately and the results traced in tables including mean, standard deviation and percentage points separating area were concerned. The status of each hospital according to the standards required under study was considered.

Mean of selected hospitals in critical standards have shown in Table 3.

**Table 2:** Rating selected hospitals in separate domains

Domains	Hospital 1	Hospital 2	Hospital 3	Hospital 4	Hospital 5	Mean
A. Leadership and management (6 subdomains: A1-A)	54.7	59.2	57.5	57.4	52.4	56.2
B. Patient and public involvement (7 subdomains: B1-B7)	40.4	46.8	37.4	53.5	38.5	43.3
C. Safe evidence-based clinical practice (6 subdomains: C1-C6)	73.6	79.4	68.6	79.1	72.1	74.6
D. Safe environment (2 subdomains: D1-D2)	84.5	81.3	80.6	82.4	79.4	81.6

**Table 3:** Mean of selected hospitals in critical standard

Hospitals	Critical standards	Mean	standard deviation	Maximum	Minimum
Hospital1	84.6	83.6	3.6	85	82.0
Hospital2	84.9				
Hospital3	81.7				
Hospital4	85.0				
Hospital5	82.0				

Critical standards of hospitals were analyzed based on the areas of leadership and administration, patient and public participation and safe evidence-based clinical practice, secure environment with and continuing education in a safe and secure environment.

As can be inferred from the above table, the average standards between surveyed hospitals did not get the required points and none of the hospitals did not include in the 4 levels (1 = highest, 4 = lowest level of classification) determined by Patient Safety Friendly Hospital Initiative.

The finding of 5 area examined showed that some components in critical standards have disadvantages (Table 4).

**Table 4:** Disadvantages of critical standards in selected hospitals

Critical Standard	Domain	Sub domain	Component	Mean	standard deviation	sig
Leadership and management	Senior management	The hospital has a patient safety programm.	76.7	3.0	0.1
		The leadership and governance are undertaking to patient safety culture.			
	Equipment	Having obligatory functioning equipment and supplies to transmit its services.	88.8	5.6	0.3
		Sterilization of equipment according guidelines			
Patient and public involvement	Health status	Informed consent	46.4	4.2	0.06
	Identify the patient	Identify the patient with two Identifier Before treatment procedure	66.7	3.0	0.1
Safe evidence-based clinical practice	General dimensions of safe clinical practice	Assurance of free communication channels for announcement the tests results	90.4	5.1	0.1
		Being procedures for announcement the delayed tests results to patients			
	Acquired infection system	Assurance of sterilization of all equipment by high risk units	93.4	3.1	0.2

## 4. Discussion

Evaluating of patient safety indicators by using the Patient Safety Friendly Hospital Initiative showed that status of the surveyed hospitals were weak based on the critical parameters according to the guideline.

In general, the total mean score of surveyed hospitals regarding the Patient Safety Friendly Hospital Protocol were lower than the gold standard.

The domain of patient and public involvement with a mean score of %.43.3 ± 4.9, which was estimated low in the surveyed hospitals. Our findings are approved with the study conducted by Najafpour et al. (2014) which found that hospital status according to patient safety standards in this domain was at a low level (42.3%) and in Siddiqi and colleagues’ study patient and public involvement (25%) had met the lowest standards. In order to achieve desired status in patient and public involvement domain, developing comprehensive programs and practical researches to hospital makes available health notice for its patients and caregivers to authorize them to share in taking the right decisions about their care, seems necessary.

And also Christian et al. (2006) confirms our findings because their study indicated that problems in communication and information stream, patient misidentification and workload were found to have negative effect in patient safety and team performance.

The domain of safe environment with a mean score of %.81.6 ± 6.8, that was estimated high in the selected hospitals. These results are also in accordance with the main findings of Najafpour study’s that hospital status according to patient safety standards in this domain was at a high level (81.2%). Also In Siddiqi and colleagues’ study safe environment (64%) was at high level (Siddiqi et al., 2012).

On the one hand, it can clarify the status of safety culture in hospitals and its strong and weak points for the managers and supervisors; and on the other hand, it has the ability to increase employees’ knowledge with respect to patient safety and assist in the improvement of the patient’s outcomes (Sahebalzamani & Mohammady, 2014; Fajardo-Dolci et al., 2010)., In Fajardo’s study like this study organizational learning items got the highest scores (Baghaee et al., 2012; Fajardo-Dolci et al., 2010). Also In Baghaee`s study the management support from patient safety was an important factor (Baghaee et al., 2012).

De Vries and colleagues in a formal search reviewed eight studies including a 74 485 patient records and reported average of adverse events events during hospital admission was 9.2% (one out of 10 patients) which 43.5% of them preventable. Substantial part of these events is preventable. A substantial part of these events were drug-related or operation (Vries, Ramrattan, Smorenburg, Gouma, & Boermeester, 2008).

An effective implementation of patient safety initiatives may depend on the legal infrastructure and enforcement of standards by hospital management, creating an organizational responsiveness to expectation of patients, creating a safety culture in hospitals and participation of patients and their families.

Although certain measures may not be reliable for international comparisons of data quality that may vary between different countries, different coding may lead to a systemic problem in some countries and communities. It needs to coordinate international efforts for increasing the accuracy of all information and documentation in hospital databases to facilitate comparative analysis. Results from the study showed that possibility of conduction and measure the change in the patient safety indicators. Our challenge is how to recognize these changes and how we interpret them.

This is the first study that addressed patient safety using by Protocol of Patient Safety Friendly Initiative (PSFHI) in Semnan hospitals. Although, the study has some limitations. First, the researchers tried to include a accountability sample of hospitals in this study as much as possible. Another limitation was inadequate cooperation from managers with interviewer and refusal to provide the information needed.

## 5. Conclusion

The findings of this study showed the PSFHI model is a useful instrument for survey of patient safety in hospitals, all the surveyed hospitals regarding standards based on patient safety had the poor condition and the identified weak points are same in the hospitals. Therefore, In order to achieve a good level of all aspects of the protocol, the goals should be considered in the level of strategic planning in hospitals.

## References

[ref1] Abdi J, Maleki R (2011). Staff Perceptions of Patient Safety Culture in Select Hospitals of Tehran University of Medical Sciences. Journal of the Iranian Institute for Health Sciences Research. Health Monitor (Payesh).

[ref2] Baghaee R, Nourani D, Khalkhali H, Pirnejad H (2012). Evaluating patient safety culture in personnel of academic hospitals in Urmia University of Medical Sciences. Bimonthly Journal of Urmia Nursing And Midwifery Faculty.

[ref3] Bates D (2001). Reducing the Frequency of Errors in Medicine Using Information Technology. Journal of the American Medical Informatics Association.

[ref4] Bates D (2003). Patient Safety:Improving Safety with Information Technology. The New England Journal of Medicine.

[ref5] Christian C. K (2006). A prospective study of patient safety in the operating room. Surgery.

[ref6] Classen D. C (2011). Global trigger tool’ shows that adverse events in hospitals may be ten times greater than previously measured. Health Affairs (Millwood).

[ref7] Edwin A (2009). Non-Disclosure of Medical Errors an Egregious Violation of Ethical Principles. Ghana Medical Journal.

[ref8] Evanoff B, Potter P, Wolf L, Grayson D, Dunagan C, Boxerman S (2005). Priorities for Patient Care Differed Among Health Care Providers. Advances in Patient Safety:From Research to Implementation. (Volume 1:Research Findings).

[ref9] Fajardo-Dolci G, Rodríguez-Suárez J, Arboleya-Casanova H, Rojano-Fernández C, Hernández-Torres F, Santacruz-Varela J (2010). Patient safety culture in healthcare professionals. Cirugia y cirujanos.

[ref10] Hashjin A. A, Kringos D. S, Manoochehri J, Ravaghi H, Klazinga N. S (2014). The associations between implementation of patient safety and patient-centeredness strategies and the type of hospitals. BMC Health Services Research.

[ref11] (2011). Iranian Ministry of Health and Medical Education (MOHME).

[ref12] Johnstone M. J, Kanitsaki O (2007). Clinical risk management and patient safety education for nurses:A critique. Nurse Education Today.

[ref13] Kohn L. T, Corrigan J. M, Donaldson M. S (2000). To err is Human:Building a Safer Health System.

[ref14] Moghri J, Arab M, Saari A. A, Nateqi E, Forooshani A. R, Ghiasvand H, Goudarzi R (2012). The psychometric properties of the Farsi version of hospital survey on patient Safety culture in Iran’s hospitals. Iranian Journal of Public Health.

[ref15] Najafpour Zh, Goudarzi Z, Keshmiri F, Pourreza A (2014). Comparison of the Education and Research Indicators of Patient Safety Status between Selected Hospitals of Tehran University of Medical Sciences Based on the WHO standards. Bimonthly journal Education Strategies Medical Sciences.

[ref16] Reynard J, Renolds J, Stevenson P (2009). Practical patient safety.

[ref17] Sahebalzamani M, Mohammady M (2014). A study of patient safety management in the framework of clinical governance according to the nurses working in the ICU of the hospitals in the East of Tehran. Iranian Journal of Nursing and Midwifery Research.

[ref18] Sari A. A, Sheldon T. A, Cracknell A, Turnbull A (2007). Sensitivity of routine system for reporting patient safety incidents in an NHS hospital:Retrospective patient case note review. BMJ.

[ref19] Siddiqi S (2012). Patient Safety Friendly Hospital Initiative:From evidence to action in seven developing country hospitals. International Journal for Quality in Health Care.

[ref20] Vries E. N, Ramrattan M. A, Smorenburg S. M, Gouma D. J, Boermeester M. A (2008). The incidence and nature of in-hospital adverse events:A systematic review. Quality and Safety in Health Care.

[ref21] WHO (2011). Assessment of Patient Safety in Hospitals:A manual for evaluators.

